# When those who benefit most participate least: socioeconomic inequalities in the well-being returns to civic engagement

**DOI:** 10.3389/fpsyg.2026.1679684

**Published:** 2026-01-15

**Authors:** Xuan Ning, Tianzhu Nie, Yi Wang

**Affiliations:** Department of Social Sciences, Beijing Normal-Hong Kong Baptist University, Zhuhai, China

**Keywords:** association membership, happiness, life satisfaction, mental health, social economic status

## Abstract

**Introduction:**

Voluntary associations are widely recognized as important sources of social connection and psychological well-being. Yet little is known about whether individuals from different socioeconomic status (SES) groups benefit equally from participation.

**Methods:**

Using data from the 2020 Canadian World Values Survey (*n* = 3,951), this study examines both (a) SES differences in association membership and (b) whether the psychological returns to membership vary across income, occupational class, and education.

**Results:**

Results show that individuals with higher SES are substantially more likely to belong to voluntary associations. However, the mental-health benefits of membership are not evenly distributed. Working-class respondents experience significantly larger gains in happiness and life satisfaction from additional memberships than service-class. Individuals with lower income reap more life satisfaction but not happiness from joining more associations, whereas people with less formal education gain more happiness but not life satisfaction than their higher-educated counterparts from joining more associations.

**Discussion:**

These findings reveal a mismatch: the groups that stand to gain the most from association participation are those least likely to join. Policies that reduce financial, informational, and structural barriers to participation may therefore yield disproportionate well-being benefits for disadvantaged populations.

## 1 Introduction

Social connectedness is a central determinant of mental well-being and community resilience (Fenn et al., 2024). Voluntary associations, such as sports clubs, cultural groups, political organizations, and neighborhood committees, provide structured spaces where individuals interact, develop shared identities, and cultivate social support (Helliwell et al., 2016; Hommerich and Tiefenbach, 2018; Nyamari, 2024; Smith and Freedman, 1972). Prior research consistently links association membership to increased happiness, reduced loneliness, and higher life satisfaction (Charles et al., 2025; Kyprianides et al., 2019). These benefits are typically understood through mechanisms such as social capital formation, shared norms, and trust (Bourdieu, 1986; Son, 2024; Son and Sung, 2024).

Bourdieu’s (1986) theory of social capital suggests that access to associative life is structurally uneven. Social resources accumulate and reproduce advantages among those who already possess economic and cultural capital. Recent empirical work confirms this pattern: higher-SES groups are far more likely to participate in voluntary associations because they have greater access to time, civic skills, and established networks (Azaiez and Salot, 2025; Wang et al., 2023). Participation thus follows a familiar inequality pattern: those with more resources are better positioned to acquire even more.

What remains unclear is whether these same advantaged groups also derive the greatest psychological benefits. One line of reasoning suggests that because advantaged groups possess greater economic, cultural, and social resources, they may be better positioned to convert participation into instrumental returns such as career advancement, status enhancement, and network expansion (Hustinx et al., 2022). From this perspective, association membership could amplify both material and psychological rewards by reinforcing a sense of efficacy, recognition, and social validation among those already well endowed with resources.

An alternative perspective, however, emphasizes diminishing marginal returns to social resources. For individuals with lower SES, who often face constrained access to stable support network and institutional recognition, association membership may provide a comparatively rare source of belonging, recognition, and interpersonal trust (Callan et al., 2017; Kluegel and Smith, 1986). Participation may therefore yield larger psychological payoffs by compensating for deficits in other forms of capital. In this sense, voluntary associations may function less as instruments of accumulation for disadvantaged groups and more as sources of emotional security and social inclusion. These competing expectations raise an important question about how psychological benefits are distributed.

This study therefore examines three related issues. First, which socioeconomic groups participate more in voluntary associations? Second, how does association membership relate to happiness and life satisfaction? Third, and most importantly, do the psychological returns to membership vary across SES? By addressing these questions, the study finds a broader “mismatch” in community participation: disadvantaged groups participate less but often gain more when they do. Understanding this mismatch offers new insight into the marginal benefits of social capital and the unequal distribution of opportunities for well-being.

## 2 Literature review

### 2.1 Association membership and mental well-being

Voluntary associations play a central role in modern social life, serving as key settings for social interaction, identity formation, and civic engagement (Barber, 2021; Smith and Freedman, 1972). These groups provide structured opportunities for individuals to connect with others, pursue shared interests, and participate in collective activities. Participation in such associations supports psychological well-being by fostering belonging, increasing social support, and building trust (Gamble and Gärling, 2012; Gómez-Balcácer et al., 2023).

Membership provides regular social interaction, access to supportive networks, and opportunities to engage in meaningful activities, each of which contributes to improved psychological functioning such as socio-emotional satisfaction and sense of control (Babchuk and Booth, 1969). Studies conducted during the COVID-19 pandemic illustrate these processes particularly clearly. Vella et al. (2023) find that continuity in group membership among young adults reduced anxiety and depression by enhancing social connectedness and hope. Similarly, Camilo et al. (2024) demonstrate that shared group identities foster self-esteem, belonging, perceived control, and an overall sense of meaning, all of which are key determinants of mental health.

Association participation also facilitates engagement in both meaningful and intrinsically rewarding activities. Volunteering is one such pathway: it allows individuals to acquire skills, develop competence, and make prosocial contributions, all of which enhance psychological well-being (Akaida et al., 2024; Omoto and Snyder, 1995; Stukas et al., 2016). Even volunteers motivated primarily by extrinsic factors, such as career signaling or networking, report increased satisfaction when the tasks align with their interests or professional goals (Ahn, 2018; Muñoz-Llerena et al., 2025). Many associations also offer opportunities for skill-building, which further reinforce self-esteem and life satisfaction (Nichol et al., 2024; Oliveira et al., 2020).

These mechanisms, including social support, meaningful activity, intrinsic motivation, and personal growth, help explain why individuals who belong to multiple associations typically report higher subjective well-being. Empirical studies consistently show that the breadth and continuity of one’s association memberships amplify these positive effects, offering repeated interpersonal contact and long-term engagement that foster durable psychological gains (Chen and Zhang, 2021; Topazian et al., 2022). This body of literature suggests that membership should produce widespread benefits, but they do not tell us whether such benefits vary across different social groups.

### 2.2 Socioeconomic disparities in association membership

Although the benefits of association participation are well established, opportunities to engage in these groups are not evenly distributed (Qvist and Munk, 2018). A large literature documents that individuals with more education, higher incomes, and professional or managerial occupations are more likely to join voluntary associations (Hwang and Young, 2022; Pusztai et al., 2021). Higher educational attainment equips individuals with civic skills, organizational knowledge, and familiarity with bureaucratic systems, making it easier to participate in structured collective activities (Azaiez and Salot, 2025; Verba et al., 1995). Education also cultivates civic norms, prosocial values, and a heightened awareness of the importance of community involvement (Alfirević et al., 2023).

Income shapes participation through access to material resources, time flexibility, and stability (Niebuur et al., 2018). Individuals with financial security face fewer barriers to sustained involvement and are better positioned to allocate time to organized voluntary activities (Sherman, 2018; Smith et al., 2017).

Occupational status similarly influences participation patterns. Those in higher-status occupations, who often enjoy greater autonomy, predictable schedules, and extensive social networks, tend to be more engaged in civic and voluntary life (Dederichs, 2024; Einolf and Chambré, 2011; Wilson and Musick, 1997). By contrast, individuals in lower-status roles may face irregular work hours, exhaustion, and limited institutional connections, which constrain opportunities for meaningful participation.

These socioeconomic disparities not only shape participation rates but also influence the potential social and professional returns of membership. Individuals with higher SES are better positioned to leverage associations for networking, career advancement, and expanded social capital (Bourdieu, 1986; Boudreaux et al., 2022). Such advantages can create a cumulative cycle in which existing resources facilitate participation, and participation further reinforces resource accumulation (Brady et al., 1995), as implied by Bourdieu’s (1986) theory of capital reproduction.

### 2.3 Do SES groups benefit equally from membership?

While much research assumes that the psychological benefits of association membership apply similarly across the population, emerging scholarship suggests that benefits may vary significantly by SES. For individuals who lack alternative support systems, such as those in lower-income or working-class positions, association membership may provide crucial emotional and social resources that compensate for economic precarity or social isolation (Adedeji et al., 2023; Lee et al., 2025). Participation in associations may offer lower-SES individuals a rare opportunity to develop other-oriented motivations, experience a sense of belonging, receive mutual aid, and build interpersonal trust that might otherwise be difficult to access (Kraus et al., 2012; Lee et al., 2025). These benefits can serve as a form of “scarcity compensation,” partially offsetting deficits in other forms of capital.

However, few studies directly test whether the well-being effects of association membership vary across SES. The absence of empirical clarity on this question highlights a core puzzle: those with fewer resources may have the highest marginal psychological returns to participation, yet remain the least likely to participate. Understanding this mismatch is essential for addressing inequality in both social participation and mental well-being.

## 3 Methodology

The data employed in this study come from the 2020 Canadian portion of the World Values Survey (WVS). Data were collected in October 2020 utilizing an online panel sampling method. The target population comprised Canadian citizens and permanent residents ages 18 and older. To ensure proportional representation, the sampling framework accounted for population size variations across cities and towns. Canada was partitioned into six regions, with sampling conducted across all regions except the Northern Territories given the negligible population proportion (0.3% of Canada). The initial dataset includes a total of 4,018 respondents; listwise deletion for the variables used in the models yields an analytical sample of 3,951.^1^ The key dependent variables represent subjective well-being operationalized as happiness and life satisfaction. Happiness is a single-item four-point ordinal measure inquiring about respondents’ self-reported general happiness levels. Life satisfaction utilizes a single-item 10-point ordinal scale evaluating respondents’ subjective life satisfaction. For both items in the analysis, higher values indicate greater subjective well-being.

Although single-item measures cannot capture the full multidimensionality of these constructs, they are widely used in large-scale population surveys and have demonstrated strong validity and reliability in prior research. Single-item life satisfaction measures correlate highly with multi-item scales and show stable psychometric properties across countries and survey waves (Cheung and Lucas, 2014). Likewise, single-item happiness measures have been shown to perform similarly to longer validated instruments in predicting well-being outcomes (Lukoševičiūtė et al., 2022). Following established practice, we therefore treat these items as valid indicators of overall subjective well-being. The primary independent variable constitutes association membership, measured as the count of voluntary organizations respondents report actively belonging to presently (Curtis et al., 2001). Assessed organization types encompass sports clubs, political parties, environmental groups, and various other civic and community associations.^2^

Key independent variables include income, occupation, and education. These are the most notable variables in social stratification research, playing a crucial role in determining an individual’s socioeconomic status within social hierarchy (Nam and Powers, 1968; Reynolds and Ross, 1998). Income is operationalized using a three-category variable derived from recoding the survey’s original 10-point self-perceived income scale. Following the coding scheme developed by the WVS survey team, respondents who selected values from 1 to 3 were classified as low income, those selecting 4 to 7 as medium, and those selecting 8 to 10 as high.^3^ Occupation uses an expanded Erikson-Goldthorpe-Portocarero (EGP) class scheme (Erikson and Goldthorpe, 1992; Ganzeboom and Treiman, 1996) to incorporate the non-employed, including service class, intermediate class, working class, retired, homemaker, and student. Service class include “professional,” “higher administrative,” and “farm proprietor”; intermediate class include “clerical,” “sales,” and “skilled worker”; working class include “service worker,” “semi-skilled worker,” “unskilled worker,” “farm worker,” and “unemployed.” Education contains four levels from secondary degree or lower, post-secondary non-tertiary, tertiary, to post-graduate.

In addition, an array of relevant sociodemographic characteristics are incorporated as statistical controls, including gender, age, marital status, immigration background, citizenship status, ethnicity, and region. Table 1 shows the distributions of all the variables.

**Table 1:** Descriptive statistics (*N* = 3,951).

Variable	Mean/prop.	SD	Min.	Max.
Happiness	3.06	0.62	1.00	4.00
Satisfaction	7.05	1.80	1.00	10.00
*N* of association membership	1.15	1.61	0.00	12.00
Income				
Low	0.15			
Medium	0.73			
High	0.12			
Occupation				
Service class	0.28			
Intermediate class	0.20			
Working class	0.18			
Retired/pensioned	0.23			
Housewife not otherwise employed	0.04			
Student	0.08			
Education				
Secondary or below	0.21			
Post-secondary non-tertiary	0.23			
Bachelor	0.42			
Post-graduate	0.14			
Male	0.51	0.50	0.00	1.00
Age	46.61	16.88	18.00	93.00
Married	0.61	0.49	0.00	1.00
Immigrant	0.18	0.38	0.00	1.00
Citizen	0.96	0.20	0.00	1.00
Ethnicity				
Caucasian	0.80			
Black	0.02			
Southeast Asian	0.01			
Arabic	0.01			
South Asian	0.03			
Latin American / Hispanic	0.01			
Aboriginal / First Nations	0.01			
Chinese	0.05			
Filipino	0.01			
Other Ethnicities	0.04			
Region				
Alberta	0.09			
British Columbia	0.19			
Manitoba	0.06			
New Brunswick	0.04			
Newfoundland and Labrador	0.03			
Nova Scotia	0.04			
Ontario	0.25			
Prince Edward Island	0.01			
Quebec	0.25			
Saskatchewan	0.04			

The analysis employs Ordinary Least Squares (OLS) regression models to estimate the effects of association membership and socioeconomic status on happiness and life satisfaction. The models also include interaction terms to examine whether the impact of association membership on mental wellbeing varies across different socioeconomic groups. Weighting provided by the WVS survey team and robust standard errors were used, but results unweighted and without robust standard errors are essentially the same. In addition, variance inflation was not found.

OLS is widely used for comparable single-item ordered well-being measures and facilitates interpretation of interaction terms, and studies found that it yields consistently results with ordered logit (Milovanska-Farrington and Farrington, 2022). On the other hand, tests show that using ordered logit would violate its parallel assumption in this case, so OLS is preferred. STATA 16 was used to perform all the analyses.

## 4 Findings

Table 2 presents the impacts of association membership and socioeconomic status on mental wellbeing. Initially, without controlling for other factors (Model 2.1), increased association membership correlates positively with higher happiness levels. This positive relationship persists after taking other variables into account (Model 2.2), though the effect strength is reduced from 0.06 point of happiness level per additional association to 0.04 point. A similar pattern arises concerning associational memberships and life satisfaction, whereby more affiliations link to higher life satisfaction, regardless of introducing control variables (Models 2.3 and 2.4). Specifically, one additional association would increase the life satisfaction level by 0.09 point. Table 2 also confirms that higher socioeconomic classes are positively associated with happiness (Model 2.3) and life satisfaction (Model 2.4). Model fit is modest but typical for cross-sectional well-being models (adjusted *R*^2^ ≈ 0.16 for happiness models with controls; adjusted *R*^2^ ≈ 0.19 for life-satisfaction models with controls).

**Table 2:** The effects of association membership on mental wellbeing.

Variable	(2.1)	(2.2)	(2.3)	(2.4)
	Happy	Happy	Satisfaction	Satisfaction
Association	0.06*** (0.01)	0.04*** (0.01)	0.14*** (0.02)	0.09*** (0.02)
Income (ref: High)				
Low		−0.49*** (0.05)		−1.96*** (0.11)
Medium		−0.21*** (0.03)		−0.89*** (0.09)
Occupation (ref: service class)				
Intermediate class		−0.01 (0.03)		−0.04 (0.09)
Working class		−0.11*** (0.04)		−0.26*** (0.09)
Retired		0.1** (0.04)		0.26*** (0.1)
Housework		−0.02 (0.07)		−0.09 (0.16)
Student		0.03 (0.05)		0.08 (0.12)
Education		0.01 (0.01)		0.02 (0.03)
Male		−0.02 (0.02)		−0.08 (0.05)
Age		0** (0)		0.01*** (0)
Married		0.2*** (0.02)		0.49*** (0.06)
Immigrant		0.06 (0.04)		0.14 (0.09)
Citizen		−0.01 (0.07)		0.13 (0.17)
Ethnicity		Yes		Yes
Region		Yes		Yes
Constant	2.97*** (0.01)	2.98*** (0.1)	6.83*** (0.04)	6.82*** (0.3)
Observations	3,951	3,951	3,951	3,951
*R*-squared	0.02	0.16	0.01	0.19

Robust standard errors are in parentheses.

***p < 0.01, **p < 0.05, *p < 0.1.

Further analyses in Table 3 reveal substantial variation in association memberships across socioeconomic strata. Individuals with higher incomes, higher occupational status, and more education tend to be members of more associations, without (Models 3.1 to 3.3) or with controls (Model 3.4). Individuals situated in higher income brackets possess memberships in significantly more organizations than lower income respondents. Specifically, after adjusting for covariates, compared to the highest income bracket, those in the lowest income bracket join 0.95 fewer associations, while those in the middle-income category join 0.71 fewer. Occupational hierarchies show similar gaps, with higher service class occupations reporting markedly more memberships than intermediate, working, and non-working groups. In particular, compared to the service class, intermediate and working classes report 0.6 and 0.5 fewer affiliations, respectively, while houseworkers, retirees, and students join 0.67, 0.37, and 0.35 fewer affiliations, respectively.

**Table 3:** The effects of socioeconomic status on association membership.

Variable	(3.1)	(3.2)	(3.3)	(3.4)
Income (ref: High)				
Low	−1.17*** (0.15)			−0.96*** (0.14)
Medium	−0.82*** (0.14)			−0.71*** (0.13)
Occupation (ref: service class)				
Intermediate class		−0.82*** (0.09)		−0.6*** (0.09)
Working class		−0.79*** (0.1)		−0.5*** (0.1)
Retired		−0.8*** (0.09)		−0.37*** (0.1)
Housework		−0.92*** (0.12)		−0.67*** (0.13)
Student		−0.45*** (0.12)		−0.35** (0.14)
Education			0.25*** (0.03)	0.17*** (0.03)
Male				0.02 (0.05)
Age				−0.01*** (0)
Married				−0.05 (0.06)
Immigrant				−0.15* (0.08)
Citizen				0.11 (0.16)
Ethnicity				Yes
Region				Yes
Constant	1.84*** (0.13)	1.66*** (0.07)	0.49*** (0.07)	2.36*** (0.3)
Observations	3,951	3,951	3,951	3,951
*R*-squared	0.04	0.05	0.02	0.11

Robust standard errors are in parentheses.

***p < 0.01, **p < 0.05, *p < 0.1.

Further, education constitutes another salient predictor of membership prevalence. Each decrease in education level corresponds with joining approximately 0.17 fewer voluntary groups. Collectively, these patterns decisively indicate that socioeconomically disadvantaged segments of society (lower income, working class, less educated) join significantly fewer community and civic organizations. This membership gap emerges even after adjusting for other socio-demographics. Model fit is also modest (adjusted *R*^2^ ≈ 0.11 the model with controls).

Table 4 shows how the influence of association membership on mental wellbeing is contingent on socioeconomic status. Models 4.1 to 4.3 are on the effects on happiness, with each focusing on the interactions between association membership and income, occupation, and education, respectively, whereas Models 4.4 to 4.6 are about the effects on satisfaction, with each focusing on the same sequence of interactions.

**Table 4:** The differential effects of association membership on wellbeing across socioeconomic groups.

Variable	(4.1)	(4.2)	(4.3)	(4.4)	(4.5)	(4.6)
	Happy	Happy	Happy	Sat	Sat	Sat
Association	0.04*** (0.01)	0.04*** (0.01)	0.08*** (0.02)	0.08*** (0.03)	0.06** (0.03)	0.13** (0.05)
Income (ref: High)						
Low	−0.52*** (0.06)	−0.49*** (0.05)	−0.49*** (0.05)	−2.06*** (0.15)	−1.96*** (0.13)	−1.96*** (0.13)
Medium	−0.2*** (0.04)	−0.21*** (0.04)	−0.21*** (0.03)	−0.87*** (0.11)	−0.89*** (0.09)	−0.89*** (0.09)
Membership* Income (ref: High)						
Low	0.04 (0.03)			0.14* (0.08)		
Medium	−0.01 (0.02)			−0.02 (0.04)		
Occupation (ref: service class)						
Intermediate class	−0.01 (0.03)	−0.01 (0.04)	−0.01 (0.03)	−0.05 (0.1)	−0.09 (0.12)	−0.04 (0.1)
Working class	−0.11*** (0.04)	−0.14*** (0.04)	−0.11*** (0.04)	−0.27** (0.11)	−0.37*** (0.12)	−0.27** (0.11)
Retired	0.1** (0.04)	0.11** (0.05)	0.1** (0.04)	0.26** (0.12)	0.25* (0.13)	0.26** (0.12)
Housework	−0.02 (0.07)	−0.05 (0.08)	−0.02 (0.07)	−0.1 (0.2)	−0.11 (0.23)	−0.1 (0.2)
Student	0.03 (0.05)	0.05 (0.06)	0.03 (0.05)	0.08 (0.15)	0.13 (0.19)	0.08 (0.15)
Membership* occupation (ref: Service class)						
Intermediate class		0 (0.02)			0.04 (0.06)	
Working class		0.04** (0.02)			0.1* (0.05)	
Retired		−0.02 (0.02)			0 (0.06)	
Housework		0.04 (0.04)			−0.01 (0.13)	
Student		−0.02 (0.03)			−0.04 (0.07)	
Education	0.01 (0.01)	0.01 (0.01)	0.03* (0.02)	0.02 (0.04)	0.03 (0.04)	0.04 (0.04)
Membership* education			−0.02** (0.01)			−0.02 (0.02)
Male	−0.02 (0.02)	−0.02 (0.02)	−0.01 (0.02)	−0.08 (0.07)	−0.09 (0.07)	−0.08 (0.07)
Age	0** (0)	0** (0)	0** (0)	0.01*** (0)	0.01*** (0)	0.01*** (0)
Married	0.2*** (0.02)	0.2*** (0.02)	0.2*** (0.02)	0.49*** (0.07)	0.5*** (0.07)	0.49*** (0.07)
Immigrant	0.06 (0.04)	0.06 (0.04)	0.06 (0.04)	0.13 (0.11)	0.14 (0.11)	0.14 (0.11)
Citizen	−0.01 (0.07)	−0.01 (0.07)	−0.01 (0.07)	0.13 (0.19)	0.13 (0.19)	0.13 (0.19)
Ethnicity	Yes	Yes	Yes	Yes	Yes	Yes
Region	Yes	Yes	Yes	Yes	Yes	Yes
Constant	2.98*** (0.1)	2.98*** (0.1)	2.94*** (0.1)	6.84*** (0.3)	6.85*** (0.3)	6.77*** (0.3)
Observations	3,951	3,951	3,951	3,951	3,951	3,951
*R*-squared	0.16	0.16	0.16	0.2	0.2	0.19

Robust standard errors are in parentheses.

***p < 0.01, **p < 0.05, *p < 0.1.

Table 4 examines interactions between association membership and SES. Models 4.1–4.3 estimate interactions for happiness and Models 4.4–4.6 for life satisfaction. For happiness, income does not significantly moderate the membership effect (Model 4.1). However, occupation does: working-class respondents gain roughly 0.08 points in happiness per additional association compared with about 0.04 points for the service class (Model 4.2). Furthermore, Model 4.3 uncovers that those with higher education levels reap less happiness from being in more associations: particularly, while those with no more than secondary education gains 0.08 point more happiness from joining one more association, those who with post-graduate degrees only obtain 0.02 point more.

Regarding life satisfaction levels, Model 4.4 demonstrates that, those in the higher income categories (medium and high) will not get much satisfaction from joining more associations, those with low income does: specifically, low-income earner joining one more association would increase their life satisfaction level by 0.22 points (=0.08 + 0.14). This catch-up effect would equate their satisfaction to medium income earners after joining five more associations. Relative to the service class, the working class gains almost triple the life satisfaction (0.16 vs. 0.06 points) per additional association (Model 4.5). By contrast, as Model 4.6 shows, the effect of association membership on life satisfaction is not contingent on education.

**Figure 1:**
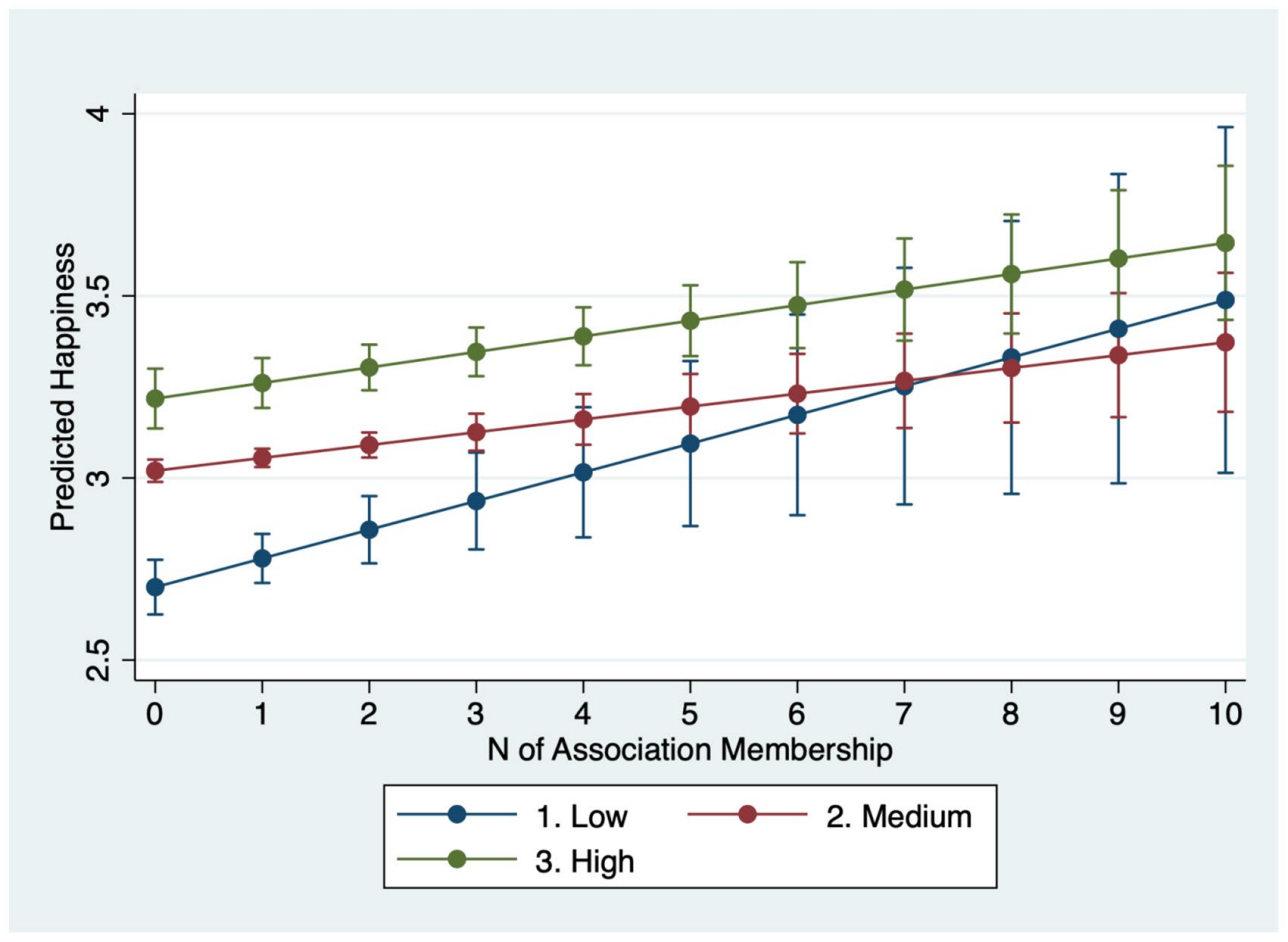
Predicted happiness by income group with 95% CIs.

**Figure 2:**
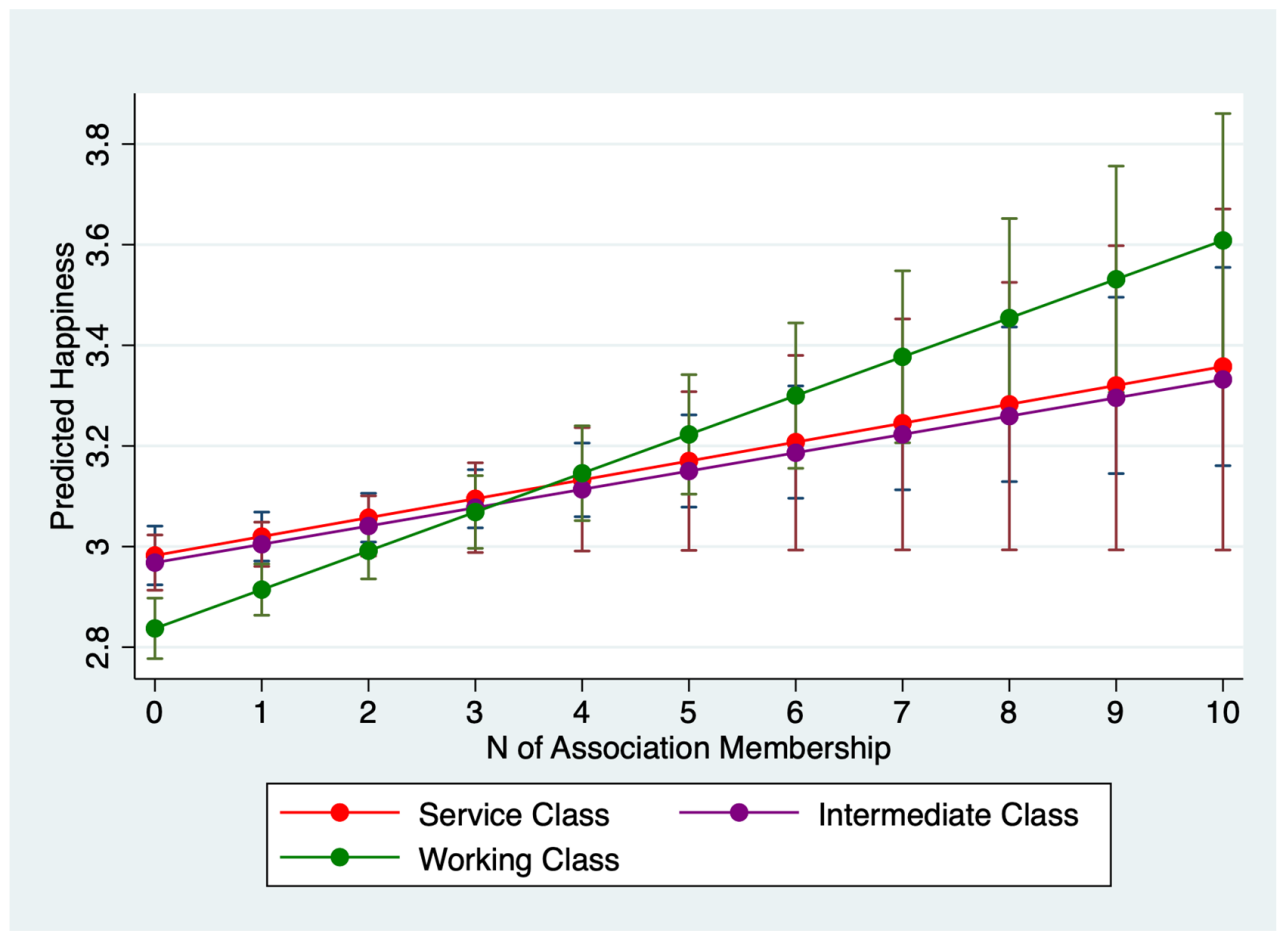
Predicted happiness by occupational group with 95% CIs.

**Figure 3:**
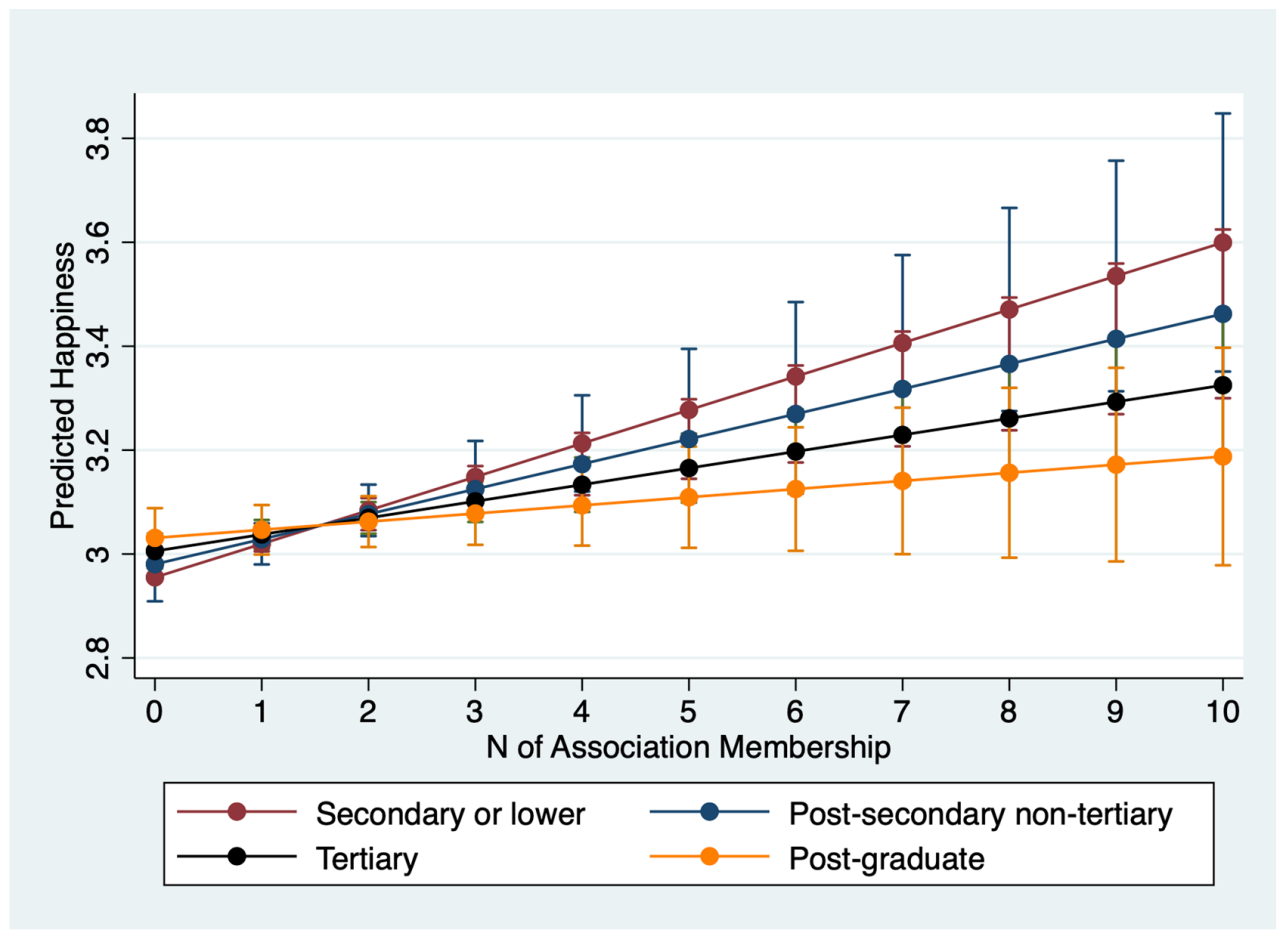
Predicted happiness by education with 95% CIs.

**Figure 4:**
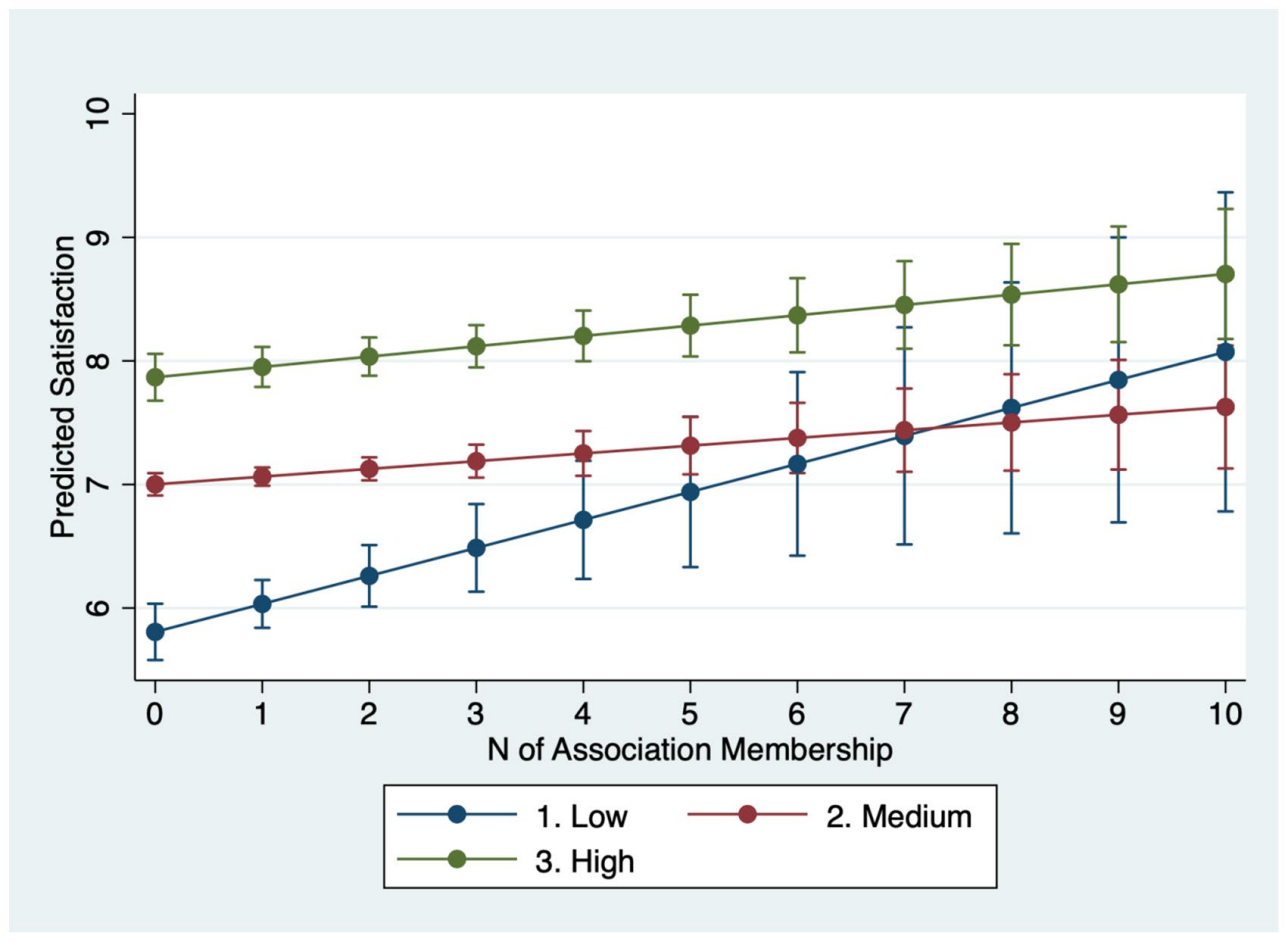
Predicted satisfaction by income group with 95% CIs.

**Figure 5:**
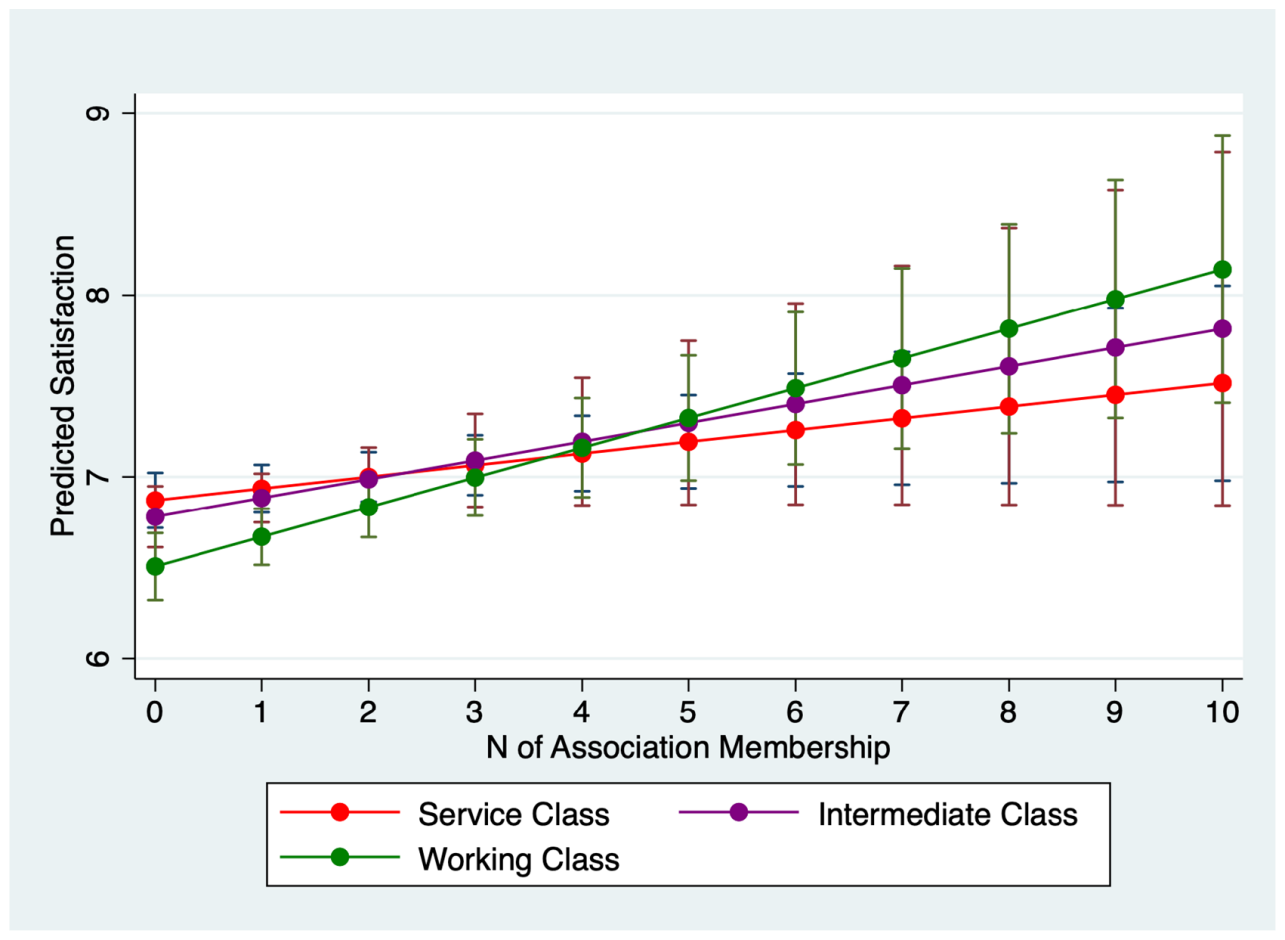
Predicted satisfaction by occupational group with 95% CIs.

**Figure 6:**
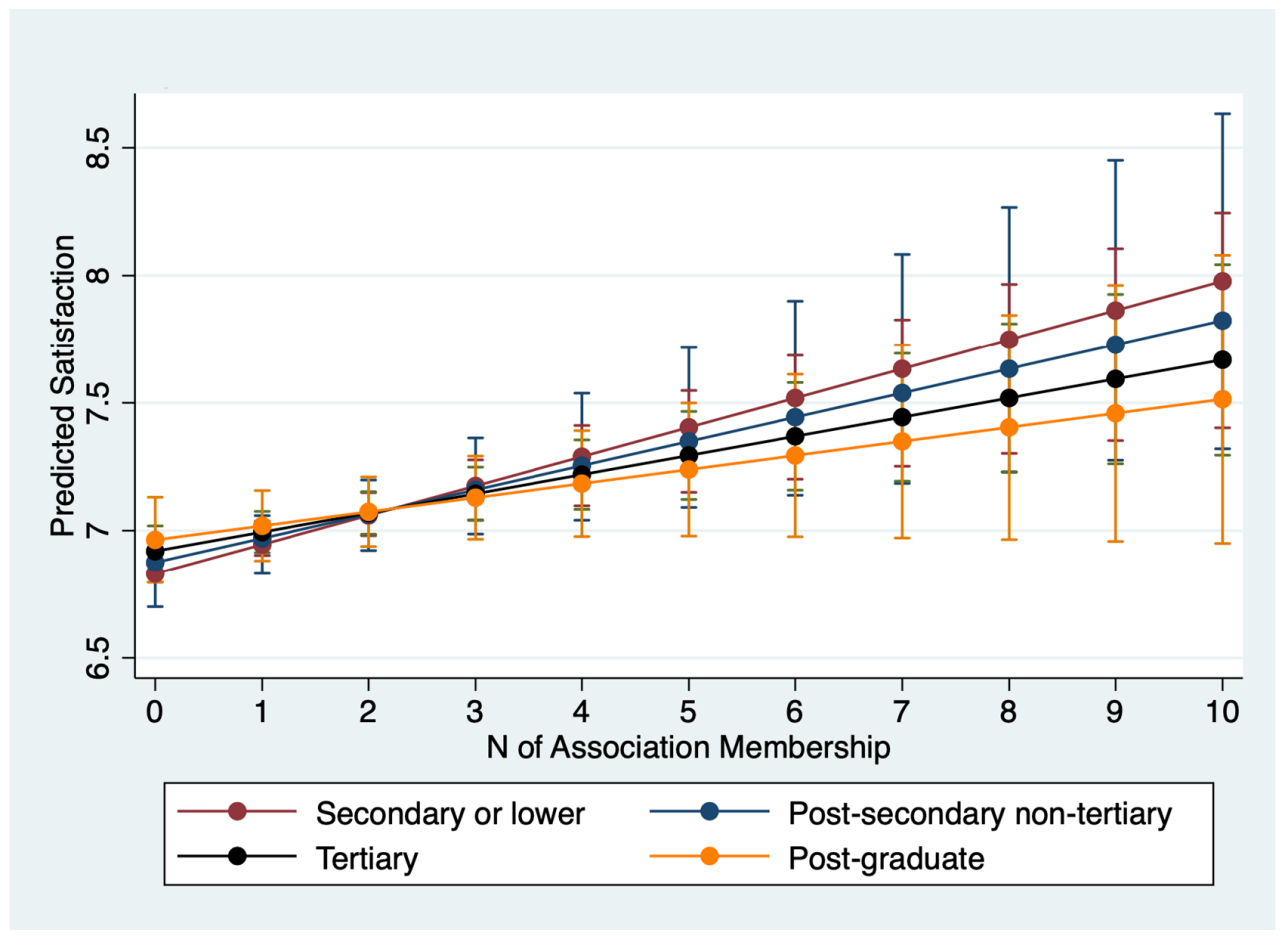
Predicted satisfaction by education with 95% CIs.

Why do income and occupation moderate life satisfaction but not always happiness? A plausible explanation lies in the psychological distinctions between the two outcomes. Life satisfaction is a global, reflective evaluation of one’s life circumstances and is therefore sensitive to structural resources and long-term psychosocial shifts that associations can provide (networks, instrumental support, social recognition) (Luhmann et al., 2012; Wang et al., 2022). Happiness (affective well-being) captures more transient mood states and may be less responsive to incremental, structural gains from association membership (Schwarz and Clore, 1983), especially in a single-item measure with limited categories. Put differently, membership appears to shift disadvantaged respondents’ evaluative judgments more than momentary affect. In summary, lower socioeconomic status individuals stand to gain greater improvements in mental wellbeing from association membership. However, they join fewer associations than their higher status counterparts. Marginal-effects plots with 95% confidence intervals (Figures 1–6) visualize these interaction dynamics and show that the slopes for Low-SES respondents are generally both steeper and statistically distinguishable from those of higher-SES groups at typical membership levels.^4^

This pattern highlights a mismatch wherein those deriving larger well-being benefits from associations partake less in them. Facilitating membership among lower socioeconomic status groups would be a promising policy lever for enhancing population mental wellbeing.

## 5 Discussion

This study set out to examine whether the psychological benefits of voluntary association membership accrue equally across socioeconomic groups. Consistent with prior research, we find that individuals with higher income, education, and occupational status are substantially more likely to participate in voluntary associations (Verba et al., 1995; Qvist and Munk, 2018). However, our central contribution lies in demonstrating that the *returns* to participation are not evenly distributed. Across multiple indicators of socioeconomic status, disadvantaged groups, particularly working-class and lower-income individuals, experience significantly larger gains in life satisfaction and, in certain cases, happiness from each additional association membership. This reveals a clear benefits–accessibility mismatch: those who stand to gain the most psychologically are precisely those who participate the least.

Placing this pattern in the context of existing research helps clarify the study’s contribution. The literature has firmly established two separate findings: first, that association membership is positively associated with subjective well-being (Helliwell et al., 2016; Charles et al., 2025), and second, that participation itself is socially stratified in favor of higher-SES groups (Azaiez and Salot, 2025; Dederichs, 2024). What has remained underexplored, however, is whether the *marginal psychological returns* to participation vary systematically across socioeconomic strata. By explicitly modeling interactions between association membership and income, occupation, and education, this study shows how social position conditions the well-being benefits of civic engagement.

In doing so, we provide empirical evidence for a pattern that has been occasionally theorized but rarely tested: that voluntary associations may function as sites of scarcity compensation for disadvantaged groups, rather than merely as mechanisms of capital accumulation for the advantaged. This finding extends debates rooted in Bourdieu’s (1986) theory of capital reproduction. While associations continue to reproduce inequality in access, our results suggest that they can simultaneously mitigate inequality in subjective well-being by generating disproportionately large benefits for those with fewer resources. In this sense, our study refines social capital theory by highlighting not only who have access, but also for whom such access matters most.

Understanding why these differential returns emerge requires attention to the social contexts in which participation is embedded. For higher-SES groups, association membership forms part of broader constellations of economic security, occupational autonomy, and dense social networks. In such contexts, individuals already possess multiple alternative sources of belonging, recognition, and control, which may limit the marginal impact of any single associational tie, yielding relatively modest additional psychological gains.

For individuals with fewer economic and institutional resources, however, association membership provides access to social goods that are otherwise scarce: stable interpersonal ties, collective identity, emotional support, and opportunities for meaningful contribution. Participation can therefore enhance life satisfaction by improving perceived social standing, buffering stress associated with economic insecurity, and fostering a sense of agency and inclusion. These mechanisms help explain why the estimated benefits are consistently larger for lower-SES respondents and why the effects are particularly strong on the evaluative dimension of well-being (life satisfaction), which captures broad assessments of life circumstances. The absence of significant moderation by income or occupation in the happiness models may reflect the more affective and momentary nature of happiness, which is typically less sensitive to structural inequalities and more influenced by temperament or daily mood fluctuations. Together, these patterns help explain why the same form of participation can generate unequal psychological payoffs across social groups.

Seen in this light, the benefits–accessibility mismatch identified here points to a clear policy opportunity. If lower-SES groups derive greater psychological returns from participation, then expanding their access to voluntary associations could yield disproportionately large improvements in population mental well-being.

One potential direction involves strengthening outreach efforts in communities where participation rates are the lowest. Partnerships with community centers, settlement organizations, public libraries, and adult learning programs can create more direct entry points for individuals who may not otherwise be exposed to voluntary groups. These organizations are already embedded in the daily lives of lower-income and working-class populations and can help introduce membership opportunities that feel both accessible and relevant.

At the same time, outreach alone is unlikely to be sufficient unless material and temporal constraints are also addressed. Sliding-scale or subsidized membership fees, childcare provision, transportation support, and flexible meeting schedules can help reduce the economic and logistical barriers that disproportionately affect lower-income individuals and those in precarious employment. Evening and weekend programming and hybrid formats can address constraints that stem from irregular employment schedules. Such efforts help ensure that participation is not limited to those with the financial stability or predictable schedules that characterize higher-SES lives.

Beyond access, the internal ethos of associations also matters. Organizations that are perceived as predominantly middle-class spaces may inadvertently discourage participation by disadvantaged groups. Actively promoting socioeconomic diversity in leadership, conducting participation audits, and creating channels for member voice such as community advisory councils can help ensure that associations are inclusive and responsive to the needs of those they seek to engage.

Finally, governments can recognize voluntary associations as part of the broader infrastructure of mental health promotion. Small operational grants, free or subsidized access to public spaces, and integrating voluntary associations into mental health promotion strategies could strengthen the capacity of associations to reach populations that stand to benefit most.

Several limitations should be acknowledged when interpreting these findings. First, the use of single-item measures of happiness and life satisfaction, while standard in large surveys and supported by prior validation studies, limits our ability to capture the multidimensional nature of subjective well-being. Future research could employ richer scales to disentangle affective, cognitive, and other dimensions. Second, although the survey uses post-stratification weights to align the sample with Canada’s regional and demographic distribution, it relies on an online panel that may underrepresent some hard-to-reach populations, which limits generalizability. Third, the cross-sectional and non-experimental design precludes causal inference. It remains possible that individuals with higher well-being are more inclined to join associations or that unobserved traits shape both. Longitudinal studies and quasi-experimental designs would be valuable in examining how changes in participation over time shape well-being trajectories across social groups and in establishing causal pathways.

Despite these limitations, the present study highlights the importance of shifting attention from who participates to who benefits most. It underscores the potential of voluntary associations not only as sites of civic engagement, but also as potential levers for reducing inequalities in subjective well-being.

## Data Availability

The datasets presented in this study can be found in online repositories. The names of the repository/repositories and accession number(s) can be found at: https://www.worldvaluessurvey.org/wvs.jsp.

## Footnotes

1 Of the 67 observations with missing values, 46 have missing values in ethnicity, 17 have missing values in education, and 2 missing values on both education and ethnicity.

2 Robustness checks using a winsorized distribution (capped at 6) produced substantively same findings (results available upon requested).

3 This recoding approach is appropriate because the measure captures respondents’ subjective assessment of their economic position rather than an objective income figure. Subjective income measures are widely used in cross-national surveys and have been shown to correlate strongly, but not perfectly, with objective income indicators (Richter and Menold, 2024). By using the survey team’s established cut-points, the coding preserves the intended interpretability of the scale while reducing noise from fine-grained self-placements that may not map meaningfully onto actual income distributions.

4 In Figures 2 and 5, three occupational categories, including retired, homemaker, and student, are omitted to highlight the key differences.
